# Tracing the rise of malignant cell lines: Distribution, epidemiology and evolutionary interactions of two transmissible cancers in Tasmanian devils

**DOI:** 10.1111/eva.12831

**Published:** 2019-06-28

**Authors:** Samantha James, Geordie Jennings, Young Mi Kwon, Maximilian Stammnitz, Alexandra Fraik, Andrew Storfer, Sebastien Comte, David Pemberton, Samantha Fox, Bill Brown, Ruth Pye, Gregory Woods, Bruce Lyons, Paul A. Hohenlohe, Hamish McCallum, Hannah Siddle, Frédéric Thomas, Beata Ujvari, Elizabeth P. Murchison, Menna Jones, Rodrigo Hamede

**Affiliations:** ^1^ School of Natural Sciences University of Tasmania Hobart Tasmania Australia; ^2^ Department of Veterinary Medicine University of Cambridge Cambridge UK; ^3^ School of Biological Sciences Washington State University Pullman Washington USA; ^4^ Department of Primary Industries, Parks, Water and the Environment (DPIPWE) Hobart Tasmania Australia; ^5^ Menzies Institute for Medical Research University of Tasmania Hobart Tasmania Australia; ^6^ Department of Biological Sciences, Institute for Bioinformatics and Evolutionary Studies University of Idaho Moscow Idaho USA; ^7^ School of Environment and Science Griffith University Nathan Queensland Australia; ^8^ Centre for Biological Sciences University of Southampton Southampton UK; ^9^ Centre for Ecological and Evolutionary Research on Cancer Montpellier France; ^10^ Centre for Integrative Ecology, School of Life and Environmental Sciences Deakin University Deakin Victoria Australia

**Keywords:** disease ecology, emerging infectious disease, epidemiology, Tasmanian devil facial tumour disease, transmissible cancer

## Abstract

Emerging infectious diseases are rising globally and understanding host‐pathogen interactions during the initial stages of disease emergence is essential for assessing potential evolutionary dynamics and designing novel management strategies. Tasmanian devils (*Sarcophilus harrisii*) are endangered due to a transmissible cancer—devil facial tumour disease (DFTD)—that since its emergence in the 1990s, has affected most populations throughout Tasmania. Recent studies suggest that devils are adapting to the DFTD epidemic and that disease‐induced extinction is unlikely. However, in 2014, a second and independently evolved transmissible cancer—devil facial tumour 2 (DFT2)—was discovered at the d’Entrecasteaux peninsula, in south‐east Tasmania, suggesting that the species is prone to transmissible cancers. To date, there is little information about the distribution, epidemiology and effects of DFT2 and its interaction with DFTD. Here, we use data from monitoring surveys and roadkills found within and adjacent to the d’Entrecasteaux peninsula to determine the distribution of both cancers and to compare their epidemiological patterns. Since 2012, a total of 51 DFTD tumours have been confirmed among 26 individuals inside the peninsula and its surroundings, while 40 DFT2 tumours have been confirmed among 23 individuals, and two individuals co‐infected with both tumours. All devils with DFT2 were found within the d’Entrecasteaux peninsula, suggesting that this new transmissible cancer is geographically confined to this area. We found significant differences in tumour bodily location in DFTD and DFT2, with non‐facial tumours more commonly found in DFT2. There was a significant sex bias in DFT2, with most cases reported in males, suggesting that since DFT2 originated from a male host, females might be less susceptible to this cancer. We discuss the implications of our results for understanding the epidemiological and evolutionary interactions of these two contemporary transmissible cancers and evaluating the effectiveness of potential management strategies.

## 1 INTRODUCTION

Emerging infectious diseases are becoming a critical concern for wildlife conservation, livestock and public health (Daszak, Cunningham, & Hyatt, 2000; Johnson, Roode, & Fenton, 2015; Jones et al., 2008). There has been an increasing interest among ecologists, evolutionary and conservation biologists in understanding and managing infectious diseases (Galvani, 2003; Johnson et al., 2015). This is because the causes and extent of local adaptations in natural host‐pathogen systems are a central aspect of adaptive evolution and species survival. Species suffering population declines from factors such as habitat loss or fragmentation are often more susceptible to emerging diseases, because of decreased genetic diversity (Altizer, Harvell, & Friedle, 2003; Ujvari et al., 2018). Initial data collection at the early stages of the epidemic onset is critical for understanding selective processes between host and pathogens and for implementing control interventions, with predictive models aiding in identifying the best management options for the disease stage (Langwig et al., 2015). For example, pathogen presence in isolated areas may allow for selective culling, aimed at reducing infection rates (Jennelle et al., 2014), or whole‐population eradication (stamping out) aimed at eliminating disease from specific geographic areas (Scudamore & Harris, 2002). However, the identification, capture and removal of a large proportion of infected individuals in wildlife diseases is in many cases not possible due to logistic constraints, particularly at large spatial scales (Langwig et al., 2015; Wobeser, 2002). A trial of selective culling to eradicate a transmissible cancer on the Forestier peninsula in south‐east Tasmania was unsuccessful (Lachish, McCallum, Mann, Pukk, & Jones, 2010), and modelling suggested that no feasible rate of culling infected animals would be successful in eradicating the disease (Beeton & McCallum, 2011).

Tasmanian devils, *Sarcophilus harrisii*, are threatened by a clonal transmissible cancer cell line–devil facial tumour disease (DFTD) (Hawkins et al., 2006; Pearse & Swift, 2006). The disease emerged more than two decades ago and has caused extensive population declines throughout 80% of the species distributional range (Lazenby et al., 2018; McCallum et al., 2009). Transmission occurs via direct inoculation of live tumour cells when devils bite each other (Hamede, McCallum, & Jones, 2013; Pearse & Swift, 2006). DFTD is able to evade host immune detection via epigenetic down‐regulation of major histocompatibility complex (MHC) gene expression in tumour cells (Siddle et al., 2013). The patterns of spatial spread observed in the first 10 years of DFTD emergence were consistent with an infectious disease from a single origin, with around 60% of the geographic range of devils in Tasmania confirmed to have DFTD by early 2007 (McCallum et al., 2007). DFTD continued to move across the landscape (Bode, Hawkins, Rout, & Wintle, 2009), but spatial spread was variable, with a rate of approximately 25 km per year southward through continuous forested habitat, compared to approximately 17 km per year southwest through heterogeneous habitat (McCallum et al., 2007). This suggests that landscape heterogeneity may influence disease spread; for example, cool temperate rainforests and highlands, both suboptimal habitat for Tasmanian devils, may act as environmental barriers (Storfer et al., 2017). At a more local scale, DFTD spread into the 160 km^2^ Freycinet peninsula on the east coast of Tasmania at a rate of only 7 km per year (McCallum et al., 2007), potentially slowed by geographic barriers such as mountains with less suitable habitat.

The epidemiology and ecology of DFTD have been consistent in all affected populations for which medium to long‐term (5 to >10 years) data are available. Given that the disease is transmitted by biting and most biting occurs during the mating season (Hamede, McCallum, & Jones, 2008; Hamede et al., 2013; Hamilton et al., 2019), DFTD transmission has been described as frequency‐dependent and affects mostly sexually mature adult devils (McCallum et al., 2009). The low DFTD prevalence in subadults might be attributed to the low number of bite wounds in this demographic group, (Hamede et al., 2013), an apparent extended latent period of DFTD (McCallum et al., 2009) and/or differences in immune function between subadults and adults (Cheng et al., 2017; Ujvari, Hamede, et al., 2016). There has been no evidence of differences in DFTD prevalence between males and females across multiple sites at different stages of the epidemic (Hamede et al., 2012; Kwon et al., 2018; Lachish, Jones, & McCallum, 2007; McCallum et al., 2009) indicating that males and females are equally susceptible to contract the disease, most likely during mating interactions. There are two ways in which DFTD transmission has been postulated, from the biter animal to the bitten (inoculation of tumour cells from infected to susceptible) or from the bitten animal to the biter (susceptible animal biting into the tumour of an infected individual). Hamede et al. (2013) found that most tumours were inside the oral cavity, suggesting that transmission occurs mostly from the bitten to the biter when the most aggressive animals deliver bites to infected subordinated individuals. Although most bite wounds have been recorded on the head, they have also been found in other parts of the body such as the rump, back, limbs and tail (Hamede et al., 2008, 2013; Hamilton et al., 2019; Pemberton & Renouf, 1993). However, almost invariably tumours have been recorded on the head and there are no records in the published literature of primary tumours occurring at any other location than the head (Hamede et al., 2013; Loh et al., 2006).

In 2014, a second clonal transmissible cancer cell line was discovered in Tasmanian devils, devil facial tumour 2 (DFT2) (Pye, Pemberton, et al., 2016). Although genetically distinct from DFTD, this newly emerged and independently evolved transmissible cancer presents similar morphological symptoms to DFTD, (Pye, Pemberton, et al., 2016). Cytogenetically, DFT2 carries a Y chromosome contrasting with the female origin of DFTD (Deakin et al., 2012; Murchison et al., 2012; Pearse & Swift, 2006; Pye, Pemberton, et al., 2016). Although sample sizes were very low, Kwon et al. (2018) reported a significant effect of sex in DFT2 prevalence. Of the 11 cases of DFT2 detected by Kwon et al. (2018), nine were males, suggesting that females could be less susceptible to this cancer. DFT2 was first reported in 2014 within the d’Entrecasteaux peninsula in south‐east Tasmania, whereas DFTD was first reported in the same peninsula in 2012 (Pye, Pemberton, et al., 2016). So far, there is limited information about the epidemiology and aetiology of DFT2 (but see Stammnitz et al., 2018) and the additional conservation threat it may pose for the already endangered species. In contrast to DFTD, DFT2 cells express MHC class I molecules, although the most highly expressed MHC alleles are shared with hosts carrying tumours (Caldwell et al., 2018). Co‐infection with DFTD and DFT2 has been reported in the same host (Kwon et al., 2018); thus, competition and selective processes between these two transmissible tumours are expected both at individual and population levels. A better knowledge of the interaction between these two transmissible cancers might allow the evaluation of potential epidemiological and evolutionary dynamics between devils, DFTD and DFT2, and assess whether management interventions are required.

Here, we determine the current distribution of DFTD and DFT2 within south‐east Tasmania, compare the population demography of the two cancers and examine the bodily location of tumours within individual hosts. We discuss the implications of our study for evaluating the epidemiological and evolutionary interactions of the two transmissible cancers and discuss the effectiveness of potential management and disease control strategies.

## 2 MATERIALS AND METHODS

### 2.1 Study area, trapping methods and data collection

**Figure 1:**
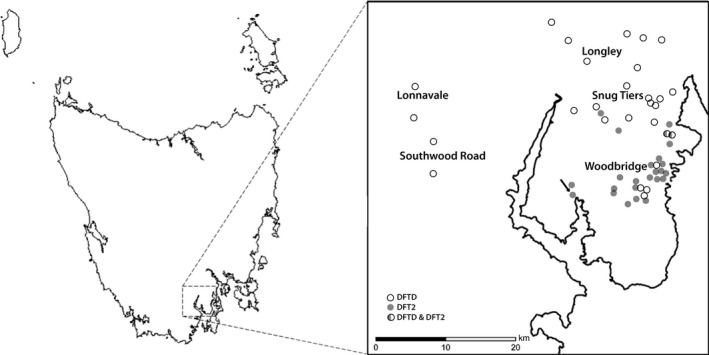
Map of Tasmania and the d’Entrecasteaux peninsula in south eastern Tasmania showing the five study sites where standard live trapping was undertaken and the location of all diseased animals. The arrow indicates the first detected case of DFT2 in 2014

Tasmanian devils were trapped at five different sites within and adjacent to the d’Entrecasteaux peninsula (43°6′S, 147°9′E), in south‐east Tasmania (Figure 1). The peninsula is located between the d’Entrecasteaux channel to the east, the Huon River to the west and the Huon Highway to the north. The area is approximately 550 km^2^ and predominantly consists of wet and dry sclerophyll forests and cleared agricultural land situated on rural residential properties along‐side crown land and nature conservation areas. The landscape is topographically varied with peaks rising to 800 m. A core monitoring area (Woodbridge, 43°09′S 147°11′E) at the centre of the peninsula was established in 2016, consisting of 40 individual trap locations covering an area of 25 km^2^. Traps were monitored for ten consecutive nights at monthly intervals during July–December 2016; July, August and December 2017; and February, April and May 2018. In addition, four adjacent trapping sites (Snug Tiers, 43°02′S, 147°10′E; Longley, 42°58′S 147°10′E; Lonnavale, 42°56′S, 146°49′E; and Southwood, 43°04′S 146°50′E) around the core area were sampled in 2011, 2015, 2016 and 2017 to monitor the presence of DFTD (2011–2018) and DFT2 (2014–2018). These sites were sampled with 40–70 traps set for five to ten nights. For a full description of trapping protocols at each site, Table S1. In all study sites, PVC culvert pipe traps were used and all captured individuals were permanently marked by implanting a microchip transponder subcutaneously at the nape of the neck. A series of standard measures including weight, sex, ageing parameters, reproductive status and disease status were collected, as described in Hamede et al. (2012). We aged devils using a combination of molar eruption, molar wear and canine over eruption (distance from the dentine–enamel junction to the gum). This method is considered precise for ageing devils up to 3 years old (Jones M., unpublished data); hence, we pooled devils of ≥3 years into a single age class. We collected biopsies for diagnosis of each individual tumour from devils trapped with visual signs of DFTD and DFT2. Tumour biopsies collected from devils that were euthanized for welfare reasons (advanced stage of DFTD/DFT2), and roadkills during 2012–2018 were also used to monitor the presence of DFTD and DFT2 (post‐2014) in the study area. Data on devil age were not available for five individuals, and therefore, these individuals were not used for the analysis of tumour type and age class. For a full list of tumour samples and metadata, see Table S2.

### 2.2 Data analysis and tumour diagnostic test

All tumours were diagnosed as DFTD or DFT2 by using the Tasman‐PCR genetic diagnostic assay (Kwon et al., 2018) or by histopathological examination of tumour biopsies (Pye, Pemberton, et al., 2016). The location of each individual tumour was recorded as either on the head (including inside the oral cavity) or on the body of the diseased animal (any location other than the head). Pearson’s chi‐square test of independence was used to evaluate differences between the location of the tumour and the disease type (DFTD or DFT2). Due to the small sample sizes across demographic groups, Fisher’s exact tests of independence were used to evaluate differences between the sex of individuals and disease type, likelihood of co‐infection, as well as the age of infected devils and disease type. All statistical analyses were performed using R (R project for Statistical Computing, version 3.4.3). The location of all infected devils was recorded and mapped in ArcGIS to map the presence of DFTD and DFT2 within and outside the d’Entrecasteaux peninsula.

## 3 RESULTS

A total of 350 individual Tasmanian devils (183 males, 163 females, one intersex and three individuals with missing data) were examined adjacent to and within the d’Entrecasteaux peninsula between 2012 and 2018. Of these, 26 individuals were diagnosed with DFTD, 23 with DFT2 and two additional individuals were co‐infected with both tumours. Of all infected devils, 13 individuals with DFTD and 10 individuals with DFT2 had more than one tumour, although co‐infection with DFTD and DFT2 in the same host was found in only two individuals (Kwon et al., 2018). This is the expected number of co‐occurrences if infection by DFTD and DFT2 is independent events (*p = *1, Fishers exact test), when analysing the entire data set. However, given that males are significantly more likely to be infected by DFT2 (see below) and that juveniles are rarely observed with tumours, we restricted the analysis to adult males. The non‐significant results (*p = *0.47, Fisher’s exact test) support the independent nature of DFTD and DFT2 infections. Since 2012, a total of 91 tumours have been confirmed in 51 individuals (51 DFTD and 40 DFT2 tumours) within the region of south eastern Tasmania, both adjacent to and within the peninsula (Figure 1). DFTD has been detected only 15 km south into the peninsula since it was first observed in 2012. Since the discovery of DFT2 in 2014, all cases have been confined to the d’Entrecasteaux peninsula (Figure 1).

**Figure 2:**
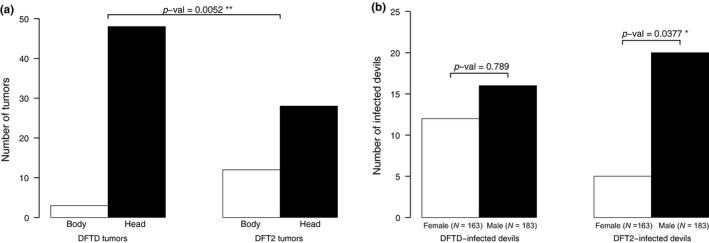
(a) Number of tumours on diseased Tasmanian devils from DFTD and DFT2 on bodily locations and (b) differences between sex and tumour type

**Figure 3:**
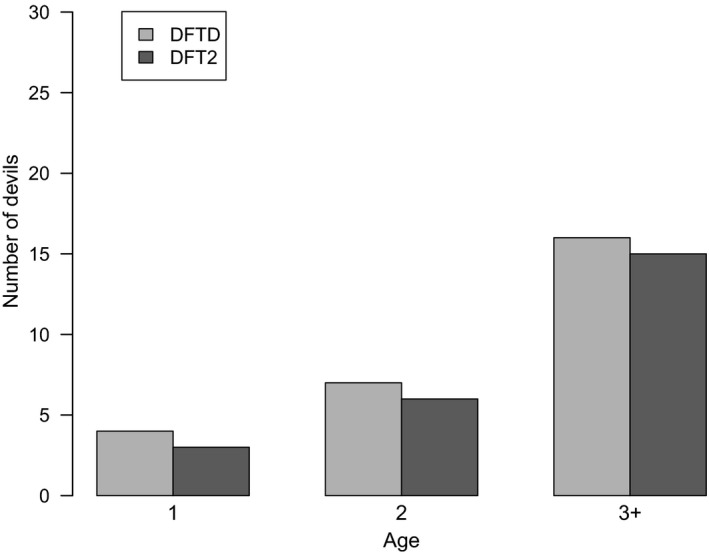
Number of Tasmanian devils infected per age class (in years) with DFTD and DFT2

There was a significant difference in the bodily location of individual DFTD and DFT2 tumours on the animals, with DFT2 tumours occurring more frequently on the body than DFTD tumours (*X* ^2^ = 7.801, *df* = 1, *p = *0.005, Figure 2a). We found a significant difference in the sex of the animal and tumour type, with more DFT2 cases in males (*p* = 0.0377, Fisher’s exact test) but this was not the case for DFTD (*p = *0.789, Fisher’s exact test) where males and females were equally affected (Figure 2b). There was no significant difference between the age of infected devils and disease type (*p = *0.99, Fisher’s exact test, Figure 3) suggesting that disease type does not affect the age at which individuals become infected.

For information on the location of animals with DFTD and DFT2 that were trapped, sampled as roadkill or euthanized and a full description of devil and tumour data, Table S2.

## 4 DISCUSSION

To date, DFT2 has been only found within the d’Entrecasteaux peninsula with all confirmed cases occurring since 2014 present in this area. As the peninsula is bounded by water on three sides, the Huon River to the west and d’Entrecasteaux Channel to the east and south, the only direction for DFT2 to escape its current geographic confinement is northward. We acknowledge that a larger sampling effort in areas north of the peninsula is necessary to firmly conclude that DFT2 has not escaped the confinement of the peninsula; therefore, our estimates of current distribution should be interpreted with caution. Continual monitoring in areas adjacent to the peninsula, particularly north of the Huon Highway and west of the Huon River are required to establish potential spread of DFT2 beyond its current known distribution. Since it was first detected in 2014, DFT2 has been found 12 km north and 16 km east from its original sampling location in the east central part of the peninsula. Although we cannot precisely identify the location of the index case of DFT2, the spatial movement at a local scale since first detection appears to be similar to the 7 km per year rate of movement of DFTD observed on the Freycinet peninsula (McCallum et al., 2007). However, at a large spatial scale, the DFT2 movement since 2014 is much slower compared to DFTD spread throughout continuous suitable habitat from its emergence in Tasmania’s north‐east in 1996, which was 25 km per year (McCallum et al., 2007). Whether this is the result of gradual spread from a single focal population or multiple populations remains unknown.

The slow movement of DFT2 across the landscape and relatively low infection rates (25 infected individuals detected, from 350 examined in 4 years, compared with DFTD prevalence of up to 50% reported by McCallum et al., 2009) may alternatively be due to interactions and/or competition with DFTD; however, this competition might have different modalities. For example, the timing of DFT2 emergence in an area already affected by DFTD and its subsequent population decline could slow the epidemic progress of DFT2. The latent period of both cancers is currently unknown, but in DFTD, it can be as long as 13 months (Save the Tasmanian devil Program, personal communication). Therefore, it is possible that infected individuals with DFTD had been subsequently infected with DFT2 but they may have succumbed to the first infection before developing visible DFT2 tumours, or vice versa.

Another plausible explanation is the difference in MHC class I expression between both tumours. There is evidence that DFT2 cells express classical and non‐classical MHC alleles, which is likely to lessen the immunogenicity of tumour cells and reduce susceptibility infection (Caldwell et al., 2018). However, the expression of MHC molecules in DFT2 is not uniform and, given its early stage of evolutionary history, is possible that it might be gradually lost, facilitating a possibly higher rate of transmission. In DFTD, it has been shown that tumour cells lack expression of MHC class I; however, this is reversible upon treatment with the inflammatory cytokine IFNγ (Siddle et al., 2013). This suggests that during the early stages of DFTD evolutionary history, MHC class I could have been expressed and subsequently down‐regulated, increasing its ability to transmit and spread throughout Tasmania. Given that DFTD was first detected in 1996 but consistent monitoring did not start until 2004, it is not possible to determine the MHC class I expression profiles of early DFTD lineages or establish whether the transmission rate of these lineages was slower than the current tumours. Further evaluation of these processes as the DFT2 epidemic unfolds will be essential to contrast the epidemiology and evolution of both cancers as well as understanding the adaptive potential of malignant cells and their hosts.

The effect of multiple tumours on disease‐induced mortality could result in a temporal reduction of the infectious period and consequently the transmission and spread of both cancers. Devils usually succumb to DFTD within 12 months after the onset of clinical signs as a result of growing tumours that lead to metabolic starvation, organ failure and in some cases metastasis (Hamede et al., 2012; Loh et al., 2006). Co‐infection with both DFTD and DFT2 has been observed in two occasions (Kwon et al., 2018) but several infected individuals in this study (25% for both DFTD and DFT2) have been observed with multiple tumours, which may result in higher metabolic demands and faster mortality rates as tumour load increases (Ruiz‐Aravena et al., 2018; Wells et al., 2017). Multiple tumours belonging to the same clone within a single host could represent (a) separate infections from different donor tumours, (b) separate infections from the same donor tumour or (c) within‐host metastases from a single infection. Although in this study we were not able to resolve these alternatives, future work on genomic sequencing of tumours may provide the necessary data to determine the origin of multiple tumours within the same host and the resulting evolutionary processes between DFTD and DFT2.

There was a significant difference in the bodily location of tumours between DFTD and DFT2, with a higher proportion of DFT2 tumours occurring more often on the body compared with DFTD. Although devil bite wounds have been recorded on the body (Hamede et al., 2013; Pemberton & Renouf, 1993), in the more than 15 years of tumour data on DFTD from multiple sites across Tasmania, non‐facial tumours have been extremely rare, with only 12 cases reported out of 1541 tumours from 574 individuals across multiple sites (Hamede R., unpublished data). Yet in this study, a third of the 40 DFT2 tumours were observed on the body. This suggests that transmission and establishment of DFT2 cells may be favouring a different niche within the host. Alternatively, DFT2 tumours on the body could be the result of metastasis from facial tumours, which could be more likely to result in external metastasis than DFTD. However, four individuals with DFT2 had tumours on the body and not on the head, indicating that non‐facial tumours can occur in the absence of facial tumours, whereas both individuals in this study with DFTD tumours on the body had facial tumours in addition to the body tumours. It is also possible that during the early evolution of DFTD non‐facial tumours were more common, but due to a reduction in transmission efficiency, they became “dead ends” and tumours that readily grew in non‐facial locations were selected against. Although non‐facial tumours are more common in DFT2, more data are needed to establish if differences with DFTD are driven by within‐host metastasis, mechanistic or selective processes.

DFT2 tumours are significantly more likely to infect male devils than female devils, differing from DFTD, which infects males and females equally, although females have been reported to have higher tolerance to DFTD infection (Ruiz‐Aravena et al., 2018). It is possible that the infection dynamics differ between DFTD and DFT2; for instance, DFT2 transmission may be more likely to occur during male–male interactions than during male–female or female–female interactions. However, studies on biting injuries and contact patterns have shown that most injuries occur between males and females during mating interactions (Hamede, Bashford, McCallum, & Jones, 2009; Hamede et al., 2013; Hamilton et al., 2019). A more plausible hypothesis is that females may have reduced susceptibility to DFT2. As DFT2 is a male cell line and carries a Y chromosome, in contrast to DFTD, which first arose from a female (Deakin et al., 2012; Murchison et al., 2012; Pye, Pemberton, et al., 2016), it is plausible that Y chromosome‐derived antigens may facilitate an immune response in female devils challenged with DFT2. This hypothesis is supported by the observation of DFT2 Y chromosome deletion in a DFT2 tumour in a female host (Stammnitz et al., 2018). If loss of the Y chromosome is a selective advantage to DFT2, then it is expected that the frequency of DFT2 cases with Y chromosome loss should increase in the near future, accompanied by a more balanced host sex ratio. If females are indeed partially resistant to DFT2, then this would reduce the basic reproductive number *R*_0 _and could account for the apparent slower rate of spread and rate of increase in prevalence, in comparison with DFTD.

We found no difference in the age of infection between DFTD and DFT2. So far, there has been no evidence that adult age classes (>2 years) differ in their susceptibility to DFTD infection. Although sample sizes are low, most infections in this study occurred in 2 and 3+ year‐old devils in both DFTD and DFT2. Since devils typically reach sexual maturity at the age of 2 years and most biting occurs in sexually mature devils during mating interactions, it is expected that these age classes would be the most affected. Previous epidemiological studies of DFTD have found higher prevalence of tumours in sexually matured adult individuals, with 1‐year‐old individuals rarely affected (Hamede et al., 2015; Lachish et al., 2007; Lazenby et al., 2018; McCallum et al., 2009). The low prevalence of DFTD and DFT2 in 1‐year‐old devils in this study is most likely due to limited mating interactions that are the primary source of bite wounds (Hamede et al., 2013; Hamilton et al., 2019). The higher prevalence of DFTD and DFT2 in sexually mature devils compared with young devils is therefore most likely driven by the higher exposure to infectious bite wounds, although immune‐driven differences in susceptibility to infection between age groups cannot be ruled out (Cheng et al., 2017; Ujvari, Hamede, et al., 2016).

Given that DFT2 was most likely detected early in its emergence, or at least at low prevalence (Pye, Pemberton, et al., 2016), and all reported cases so far have been restricted to the d’Entrecasteaux peninsula, we suggest that continual monitoring of the disease to further evaluate its spread, transmission dynamics, population effects and evolutionary trajectory should be a priority. Considering that disease eradication strategies at a large scale are not logistically feasible and that Tasmanian devils may be prone to transmissible cancers in general (Storfer et al., 2018), we suggest that conservation efforts are focused on evaluating and maintaining the adaptive genetic diversity of devils in response to the DFTD and DFT2 epidemics and their potential evolutionary dynamics.

Competition and co‐infection between DFTD and DFT2 may alter the current adaptive responses and evolutionary processes between devils and DFTD, resulting in changes in virulence and transmission dynamics in both cancers. There is increasing evidence that oncogenic phenomena have an important role in driving ecological and evolutionary processes, from individuals to ecosystems (Thomas et al., 2013, 2017; Vittecoq et al., 2018). The recent discovery of five transmissible cancers in marine bivalves (Metzger et al., 2016) suggests that malignant cell lines with the potential of becoming transmissible diseases are more common than previously thought. As Tasmanian devils have been affected by two transmissible cancers over the last 20 years, it is also possible that these types of cancers have previously appeared and subsequently died out through the species’ evolutionary history (Ujvari, Gatenby, & Thomas, 2016). How these two transmissible cancers will interact is currently unknown, and it is possible that interactions between DFTD and DFT2 may alter acquired adaptive processes that have occurred in the devil‐DFTD system for more than 20 years. These adaptations include changes in susceptibility to infection across tumour karyotypes (Hamede et al., 2015), devil immune expression profiles (Ujvari, Hamede, et al., 2016), the presence of antibodies associated with natural tumour regressions (Pye, Hamede, et al., 2016), changes in allele frequencies of genes associated with immune function in as little as four to six generations since exposure to DFTD (Epstein et al., 2016), as well as differences in tolerance and survival after infection (Margres et al., 2018; Ruiz‐Aravena et al., 2018). Molecular studies have demonstrated that DFTD is subject to adaptability and evolutionary plasticity (Deakin et al., 2012; Murchison et al., 2012; Pearse et al., 2012; Ujvari, Gatenby, et al., 2016; Ujvari et al., 2013) which may also affect epidemiology and population effects. Continual monitoring of the DFT2 epidemic and its interaction with DFTD in ecological timescales will be essential for understanding important concepts of cancer ecology and evolution. This will allow evaluation of potential genetic management strategies and assessment of the conservation threat imposed by this newly evolved transmissible cancer.

## CONFLICT OF INTEREST

None declared.

## Data Availability

Data available from the Dryad Digital Repository: https://doi.org/10.5061/dryad.jj83827.
