# Supplementary material for: Tracing the rise of malignant cell lines: Distribution, epidemiology and evolutionary interactions of two transmissible cancers in Tasmanian devils
Source: Evol Appl. 2019 Jun 28;12(9):1772–80. doi: 10.1111/eva.12831 (PMC6752152; doi:10.1111/eva.12831)
Supplement: Supplementary file 1 [file EVA-12-1772-s001.docx]

**SUPPLEMENTARY DATA Distribution and epidemiology of two transmissible cancers in Tasmanian devils.**

TABLE S1: Five trapping sites within and adjacent to the d’Entrecasteaux Channel peninsula, Australia where live trapping was undertaken. Area of each site (km^2^), location reference, time range of sampling, number of traps used and the number of nights traps were set for are provided.

Table S2: List of tumor samples and associated metadata

| **Devil microchip identifier** | **Sex** | **Age (in years)** | **Tumour number** | **Tumour location** | **Tumour diagnostic** | **Site Name** | **Sample origin** |
| --- | --- | --- | --- | --- | --- | --- | --- |
| 982000363393409 | Female | unknown | T1 | Head | DFT2 | Nicholls Rivulet | Live trapping |
| 982000167806989 | Male | 1 | T1 | Head | DFTD | Margate | Live trapping |
| 982000356432540 | Female | 3+ | T2 | Head | DFTD | Woodbridge | Live trapping |
| 982000356432540 | Female | 3+ | T1 | Head | DFTD | Woodbridge | Live trapping |
| 982000365600687 | Female | 3+ | T1 | Body | DFT2 | Woodbridge | Live trapping |
| SDD000000100315 | Male | 2 | T1 | Head | DFTD | Crabtree | Euthanised |
| SDD000000100315 | Male | 2 | T2 | Head | DFTD | Crabtree | Euthanised |
| SDD000000100315 | Male | 2 | T3 | Head | DFTD | Crabtree | Euthanised |
| Unknown | Female | 2 | T1 | Head | DFTD | Crabtree | Roadkill |
| 982000356584128 | Female | 2 | T1 | Head | DFTD | Woodbridge | Live trapping |
| 982000402727720 | Female | 3+ | T2 | Body | DFT2 | Woodbridge | Live trapping |
| 982000402727720 | Female | 3+ | T1 | Head | DFT2 | Woodbridge | Live trapping |
| 982000365601023 | Male | 3+ | T1 | Head | DFT2 | Woodbridge | Live trapping |
| 982000365601023 | Male | 3+ | T4 | Head | DFT2 | Woodbridge | Live trapping |
| 982000365601023 | Male | 3+ | T2 | Head | DFT2 | Woodbridge | Live trapping |
| 982000365601023 | Male | 3+ | T3 | Head | DFT2 | Woodbridge | Live trapping |
| 982000356444429 | Female | 3+ | T1 | Head | DFTD | Grove | Euthanised |
| 982000356444429 | Female | 3+ | T2 | Head | DFTD | Grove | Euthanised |
| 982000356444429 | Female | 3+ | T3 | Head | DFTD | Grove | Euthanised |
| 982000365119580 | Male | 3+ | T1 | Head | DFTD | Southwood Road | Live trapping |
| 982000405797721 | Female | 3+ | T2 | Body | DFTD | Longley | Live trapping |
| 982000405797721 | Female | 3+ | T1 | Head | DFTD | Longley | Live trapping |
| 982000356669303 | Male | 2 | T1 | Head | DFT2 | Snug Tiers | Euthanised |
| 982000363452012 | Male | 2 | T2 | Head | DFT2 | Nicholls Rivulet | Euthanised |
| **Devil microchip identifier** | **Sex** | **Age (in years)** | **Tumour number** | **Tumour location** | **Tumour diagnostic** | **Site of capture** | **Sample origin** |
| 982000363452012 | Male | 2 | T1 | Head | DFT2 | Nicholls Rivulet | Euthanised |
| 9820003656710850 | Female | 3+ | T3 | Head | DFTD | Snug Tiers | Live trapping |
| 9820003656710850 | Female | 3+ | T2 | Head | DFTD | Snug Tiers | Live trapping |
| 9820003656710850 | Female | 3+ | T1 | Head | DFTD | Snug Tiers | Live trapping |
| 982000356577185 | Male | 1 | T1 | Head | DFTD | Woodbridge | Live trapping |
| S0000000006148 | Male | 1 | T1 | Head | DFT2 | Kettering | Carcass |
| 982000365611366 | Male | 3+ | T4 | Body | DFT2 | Woodbridge | Live trapping |
| 982000365611366 | Male | 3+ | T2 | Body | DFT2 | Woodbridge | Live trapping |
| 982000365611366 | Male | 3+ | T3 | Body | DFT2 | Woodbridge | Live trapping |
| 18/2221 | Male | unknown | T1 | Head | DFTD | Leslie Vale | Carcass |
| 18/2221 | Male | unknown | T2 | Head | DFTD | Leslie Vale | Carcass |
| 18/2221 | Male | unknown | T5 | Body | DFTD | Leslie Vale | Carcass |
| 18/2221 | Male | unknown | T6 | Body | DFTD | Leslie Vale | Carcass |
| 982000356483454 | Female | 3+ | T3 | Head | DFTD | Snug | Live trapping |
| 982000356483454 | Female | 3+ | T1 | Head | DFT2 | Snug | Live trapping |
| 982000356483454 | Female | 3+ | T2 | Head | DFTD | Snug | Live trapping |
| D000000150717 | Male | 2 | T1 | Head | DFTD | Margate | Carcass |
| D000000150717 | Male | 2 | T2 | Head | DFTD | Margate | Carcass |
| D000000150717 | Male | 2 | T3 | Head | DFTD | Margate | Carcass |
| D000000150717 | Male | 2 | T4 | Head | DFTD | Margate | Carcass |
| D000000150717 | Male | 2 | T5 | Head | DFTD | Margate | Carcass |
| 982000365595466 | Male | 3+ | T1 | Head | DFTD | Southwood Road | Live trapping |
| 982000365596017 | Male | 2 | T1 | Body | DFT2 | Woodbridge | Live trapping |
| 982000365596017 | Male | 2 | T2 | Body | DFT2 | Woodbridge | Live trapping |
| 982000363454219 | Female | 2 | T1 | Head | DFTD | Pelverata | Live trapping |
| 982000363454219 | Female | 2 | T2 | Head | DFTD | Pelverata | Live trapping |
| **Devil microchip identifier** | **Sex** | **Age (in years)** | **Tumour number** | **Tumour location** | **Tumour diagnostic** | **Site of capture** | **Sample origin** |
| Unknown | Female | 2 | T1 | Head | DFT2 | Oyster Cove | Roadkill |
| 982000402727994 | Male | 3+ | T1 | Body | DFT2 | Woodbridge | Live trapping |
| 982000402727994 | Male | 3+ | T2 | Body | DFT2 | Woodbridge | Live trapping |
| 982000402727994 | Male | 3+ | T3 | Body | DFT2 | Woodbridge | Live trapping |
| 982000405826904 | Male | 3+ | T1 | Head | DFTD | Longley | Live trapping |
| 982000190608331 | Male | 3+ | T1 | Head | DFT2 | Cygnet | Live trapping |
| 982000402222045 | Male | 3+ | T1 | Head | DFT2 | Woodbridge | Live trapping |
| 982000402222045 | Male | 3+ | T2 | Head | DFT2 | Woodbridge | Live trapping |
| 982000361951310 | Female | 3+ | T1 | Head | DFTD | Margate | Live trapping |
| R00000000002968 | Male | 3+ | T1 | Head | DFTD | Sandfly | Roadkill |
| 982000361974890 | Male | 3+ | T1 | Head | DFTD | Woodbridge | Live trapping |
| 982000361974890 | Male | 3+ | T2 | Head | DFTD | Woodbridge | Live trapping |
| 982000167763080 | Male | 3+ | T1 | Head | DFT2 | Nicholls Rivulet | Live trapping |
| 982000365112286 | Female | 3+ | T1 | Head | DFTD | Southwood Road | Live trapping |
| 982000405909352 | Female | 3+ | T1 | Head | DFTD | Snug Tiers | Live trapping |
| 982000365120134 | Male | 2 | T2 | Head | DFT2 | Woodbridge | Live trapping |
| 982000405826357 | Male | 3+ | T1 | Head | DFT2 | Woodbridge | Live trapping |
| 982000356526917 | Male | 3+ | T1 | Head | DFT2 | Snug | Euthanised |
| 982000356526917 | Male | 3+ | T2 | Head | DFT2 | Snug | Euthanised |
| 982009104799360 | Female | 3+ | T1 | Head | DFTD | Snug | Euthanised |
| 982000356711103 | Male | 1 | T1 | Head | DFTD | Snug Tiers | Euthanised |
| 982000405792676 | Male | 3+ | T3 | Body | DFT2 | Woodbridge | Live trapping |
| 982000405792676 | Male | 3+ | T1 | Head | DFT2 | Woodbridge | Live trapping |
| 982000405792676 | Male | 3+ | T2 | Body | DFT2 | Woodbridge | Live trapping |
| R00000020151130 | Male | 3+ | T1 | Head | DFT2 | Cygnet | Roadkill |
| Unknown | Male | 2 | T1 | Head | DFT2 | Woodbridge | Carcass |
| **Devil microchip identifier** | **Sex** | **Age (in years)** | **Tumour number** | **Tumour location** | **Tumour diagnostic** | **Site of capture** | **Sample origin** |
| 982000167740522 | Male | 1 | T1 | Head | DFT2 | Snug | Live trapping |
| 982000167740522 | Male | 1 | T2 | Head | DFTD | Snug | Live trapping |
| 982000361993284 | Male | 3+ | T2 | Head | DFT2 | Woodbridge | Live trapping |
| 982000361993284 | Male | 3+ | T1 | Head | DFT2 | Woodbridge | Live trapping |
| 982000405851834 | Male | 3+ | T1 | Head | DFTD | Longley | Live trapping |
| 982000405851834 | Male | 3+ | T4 | Head | DFTD | Longley | Live trapping |
| 982000405851834 | Male | 3+ | T2 | Head | DFTD | Longley | Live trapping |
| 982000405851834 | Male | 3+ | T3 | Head | DFTD | Longley | Live trapping |
| S000000120116 | Male | 2 | T2 | Head | DFTD | Margate | Live trapping |
| S000000120116 | Male | 2 | T1 | Head | DFTD | Margate | Live trapping |
| 982000363392739 | Male | 3+ | T1 | Head | DFT2 | NA | Live trapping |
| 982000356577211 | Male | 2 | T2 | Head | DFTD | Woodbridge | Live trapping |
| 982000356577211 | Male | 2 | T1 | Head | DFTD | Woodbridge | Live trapping |
| 982000405911176 | Male | 3+ | T1 | Head | DFTD | Southwood Road | Live trapping |
| R00000000007370 | Male | 1 | T1 | Head | DFT2 | Woodbridge | Roadkill |
